# Supplementary material for: Characterization of genetic and molecular tools for studying the endogenous expression of Lactate dehydrogenase in Drosophila melanogaster
Source: bioRxiv. 2023 Jun 23:2023.06.15.545165. Preprint. [Version 3] doi: 10.1101/2023.06.15.545165 (PMC10312709; doi:10.1101/2023.06.15.545165)
Supplement: Supplement 1 [file media-1.pdf]

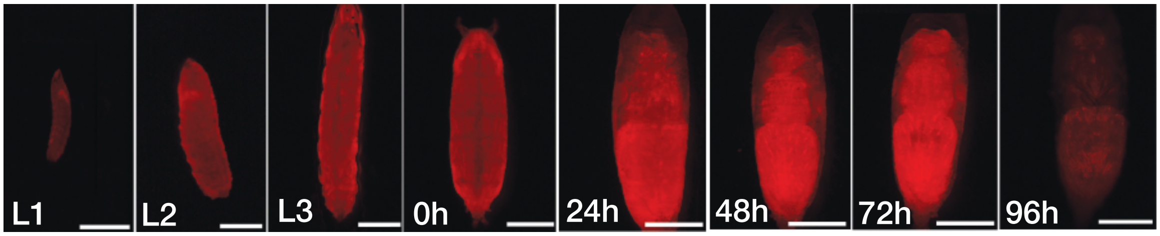

Figure S1. Expression of *Ldh-mCherry*^{Genomic} during larval development. The *Ldh-mCherry*^{Genomic} spatial expression pattern is consistent with previous studies, with Ldh-mCherry^{Genomic} being expressed at high levels in (A) the body wall muscle. However, unlike Ldh-GFP^{Genomic}, the (A) expression of mCherry^{Genomic} fusion protein persists throughout much of pupal development (compare with Figure 1B).
