# Supplementary material for: Characterization of genetic and molecular tools for studying the endogenous expression of Lactate dehydrogenase in Drosophila melanogaster
Source: bioRxiv. 2023 Jun 23:2023.06.15.545165. Preprint. [Version 3] doi: 10.1101/2023.06.15.545165 (PMC10312709; doi:10.1101/2023.06.15.545165)
Supplement: Supplement 2 [file media-2.pdf]

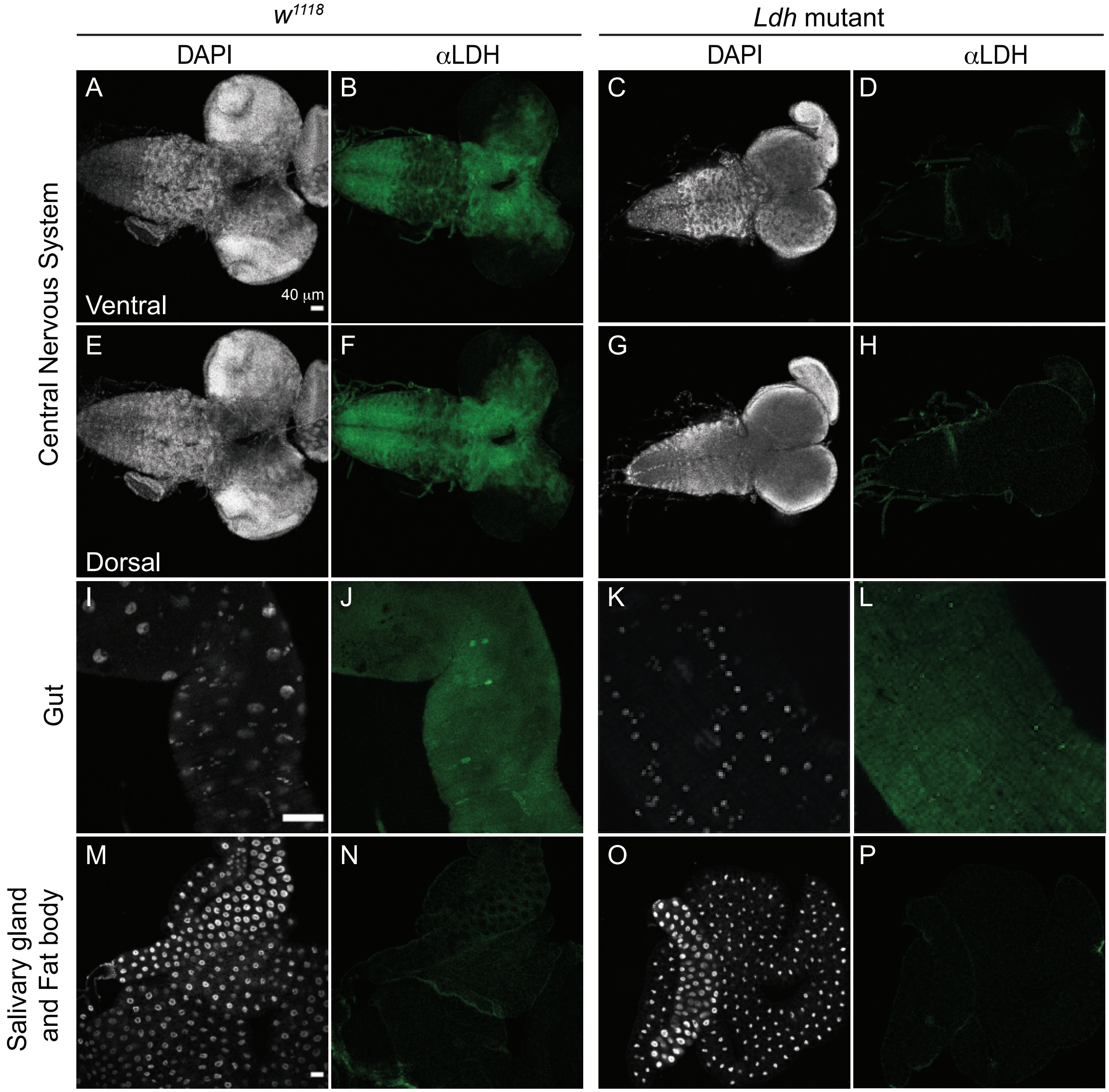

Figure S2. αLdh immunostaining in control and *Ldh* mutant larval tissues. Representative confocal images of the (A-H) central nervous system, (E-H) intestine, (I-L) fat body, and (M-P) salivary gland dissected from third instar w^{1118} and Ldh^{16} larvae. DAPI is shown in white and *Ldh* expression is shown in green. Note the brightness in (L) was increased to highlight the lack of staining in small cells within AMP clusters. The scale bar in all images represents 40 μM. The scale bar in (A) applies to panels (B-H), the scale bar in (I) applies to panels (J-L), and the scale bar in (M) applies to panels (N-P).
